# Supplementary material for: Otological Manifestations in Adults with Primary Ciliary Dyskinesia: A Controlled Radio-Clinical Study
Source: J Clin Med. 2022 Aug 31;11(17):5163. doi: 10.3390/jcm11175163 (PMC9456589; doi:10.3390/jcm11175163)
Supplement: Supplementary file 1 [file jcm-11-05163-s001.zip › jcm-1857857-supplementary.pdf]

Supplementary Table S1. Detailed genotypes of the 17 PCD [1] patients.

Phenotypic Group	Patient	Gene	Allele 1	Allele 2	Status	Variant Classification Allele 1	Variant Classification Allele 2
IDA+MTD [2]	18GM00900	*CCDC39*	c.890del p.(Tyr297Phefs*11)	c.890del p.(Tyr297Phefs*11)	Homozygous or hemizygous	Frameshift	Frameshift
IDA+MTD	17GM01057	*CCDC40*	c.248del p.(Ala83Valfs*84)	c.2712-1G>T p.?	Heterozygous x2	Frameshift	Splice
IDA+MTD	18GM02046	*CCDC40*	c.1464del p.(Ser489Alafs*18)	c.1464del p.(Ser489Alafs*18)	Homozygous	Frameshift	Frameshift
IDA+MTD	8821GM001177	*CCDC40*	c.3097A>T p.(Lys1033*)	c.3097A>T p.(Lys1033*)	Homozygous	Nonsense	Nonsense
ODA [3]	17GM02506	*DNAH5*	c.841_842insCTTCCGC p.(Val281Alafs*20)	c.841_842insCTTCCGC p.(Val281Alafs*20)	Homozygous	Frameshift	Frameshift
ODA	17GM01074	*DNAH5*	c.5290T>C p.(Ser1764Pro)	c.5290T>C p.(Ser1764Pro)	Homozygous	Missense	Missense
ODA	17GM02727	*DNAH5*	c.6614_6617del p.(Ile2205Lysfs*30)	c.11827T>G p.(Trp3943Gly)	Compouned heterozygous	Frameshift	Missense
ODA	17GM00384	*DNAL1*	c.315del p.(Ile105Metfs*4)	c.315del p.(Ile105Metfs*4)	Homozygous	Frameshift	Frameshift
2DA [4]	8821GM001180	*DNAAF1*	c.1022_1023del p.(Gln341Argfs*10)	c.1022_1023del p.(Gln341Argfs*10)	Homozygous	Frameshift	Frameshift
nEM [5]	17GM02360	*CCDC65*	c.877_878del p.(Ile293Profs*2)	c.877_878del p.(Ile293Profs*2)	Homozygous	Frameshift	Frameshift
nEM	8821GM001211	*DNAH11*	c.2569C>T p.(Arg857*)	c.4255-5A>G p.?	Compouned heterozygous	Nonsense	Splice
nEM	17GM02672	*DNAH11*	c.8524C>T p.(Arg2842Trp)	c.10073del p.(Ala3358Glufs*12)	Heterozygous x2	Missense	Frameshift
nEM	8821GM001178	*DNAH11*	c.13303+5G>A	c.13303+5G>A	Homozygous	Splice	Splice
nEM	18GM00443	*DNAH11*	c.8117A>G p.(Tyr2706Cys)	c.8117A>G p.(Tyr2706Cys)	Homozygous	Missense	Missense
nEM	17GM01640	*HYDIN*	c.6802C>T p.(Gln2268*)	c.6802C>T p.(Gln2268*)	Homozygous	Nonsense	Nonsense
nEM	17GM01863	*RPGR*	c.2149G>T p.(Gly717*)	-	Hemizygous	Nonsense	-
CC [6]	18GM00096	*RSPH9*	c.804_806del p.(Lys268del)	c.804_806del p.(Lys268del)	Homozygous	Single amino acid deletion	Single amino acid deletion

[1] primary ciliary dyskinesia; [2] inner dynein arm defect with microtubular disorganization; [3] outer dynein arm defect; [4] both dynein arms defect, [5] normal electronic microscopy, [6] central complex defect.
